# Flying under the radar: vancomycin heteroresistance in *Staphylococcus epidermidis* peritonitis is associated with treatment failure

**DOI:** 10.1128/asmcr.00007-25

**Published:** 2025-07-09

**Authors:** Malgorzata K. Kopczyk, Joshua P. Ramsay, Kieran T. Mulroney, Emily Salisbury, Wai Shaun Ho, Christine F. Carson, Elena Colombi, Teagan Paton, Aron Chakera

**Affiliations:** 1 UWA Center for Medical Research (affiliated with the Harry Perkins Institute of Medical Research), The University of Western Australia 2720, Perth, Australia; 2 UWA Medical School, The University of Western Australia 2720, Perth, Australia; 3 Curtin Health Innovation Research Institute and Curtin Medical School, Curtin University Perth, Perth, Australia; 4 School of Natural Sciences, Macquarie University, Sydney, New South Wales, Australia; 5 ARC Center of Excellence for Synthetic Biology, Macquarie University, Sydney, New South Wales, Australia; 6 Department of Renal Medicine, Sir Charles Gairdner Hospital, Perth, Australia; Pattern Bioscience, Austin, Texas, USA

**Keywords:** heteroresistance, treatment failure, antimicrobial resistance, peritonitis

## Abstract

**Background:**

Antimicrobial resistance (AMR) is a global One Health problem and a growing threat to human and animal health. Antimicrobial susceptibility tests (AST) such as the broth-microdilution method (BMD) guide appropriate antimicrobial therapy but may fail to detect various forms of AMR, such as heteroresistance. We report vancomycin treatment failure in a *Staphylococcus epidermidis* peritoneal dialysis (PD) associated peritonitis case with vancomycin heteroresistance undetected by conventional AST and whole-genome sequencing.

**Case Summary:**

A patient with cloudy PD effluent presented to his local dialysis center. Samples were sent for testing, and empirical treatment with vancomycin and gentamicin was initiated. An *S. epidermidis* isolate (C099) was cultured, and treatment was changed to vancomycin monotherapy, with the isolate recorded as susceptible using the VITEK 2. Despite treatment, effluent remained culture positive, and two additional *S. epidermidis* isolates were cultured (C100 and C101). These isolates were also recorded as susceptible to vancomycin using the VITEK 2. Seventeen days post-presentation, the PD catheter was removed due to persistent infection, and the patient was transferred to hemodialysis.

**Conclusion:**

Conventional AST (VITEK 2 and BMD) misclassified the isolates as vancomycin susceptible, while population analysis profiling area under the curve confirmed heteroresistance. Whole-genome sequencing did not identify genetic mechanisms for the heteroresistance, with no mutations detected in known resistance-associated genes. This study highlights the limitations of conventional AST and genomic methods for AMR detection, emphasizing the need for further research into cryptic forms of heteroresistance to enhance diagnostic accuracy and improve patient outcomes.

## INTRODUCTION

Peritonitis is a serious complication of peritoneal dialysis (PD) and remains associated with morbidity, treatment failure, and mortality (1). PD-associated peritonitis is most commonly caused by gram-positive bacteria (2, 3), with coagulase-negative staphylococci (CoNS) such as *Staphylococcus epidermidis* contributing to approximately half of the cases (4, 5). Compared to peritonitis caused by other micro-organisms, CoNS peritonitis has the highest rates of relapse, recurrence, and catheter removal (6, 7), which can lead to prolonged hospitalizations, further increasing the healthcare cost burden.

The International Society for Peritoneal Dialysis (ISPD) recommends the prompt use of empirical antimicrobial therapy to cover both gram-positive and gram-negative bacteria in peritonitis cases (8). In our unit, vancomycin and gentamicin are administered intraperitoneally (IP) (9) with further treatment guided by the results of culture and antimicrobial susceptibility test (AST) results. While the broth-microdilution method (BMD) is the gold-standard AST, clinical laboratories utilize automated phenotypic testing methods (such as the VITEK 2) to reduce time to result and increase throughput (10).

Antimicrobial resistance (AMR) is a serious threat to peritonitis patients, limiting effective treatment options and associated with higher treatment failure and relapse rates (11). Heteroresistance is a form of AMR where a small subset of a bacterial population exhibits increased resistance to antimicrobial agents (12, 13). It has been documented in a variety of bacterial species including CoNS (14) but can be undetected by conventional AST methods (15, 16). This can lead to the misclassification of the AMR profile of the organism, resulting in ineffective antimicrobial therapy (12).

We report heteroresistance in a multi-drug-resistant (MDR) *S. epidermidis* isolated from a PD peritonitis case. The infection did not resolve, resulting in eventual vancomycin treatment failure. Despite phenotypic AST repeatedly confirming the sensitivity of the *S. epidermidis* isolates to vancomycin, drained PD effluent remained culture positive. Using multiple phenotypic methods and whole-genome sequencing (WGS), we characterized the three *S*. *epidermidis* isolates isolated sequentially (days 2, 7, and 16) during the peritonitis episode and confirmed the presence of heteroresistance as a potential explanation for the failure to resolve infection.

## CASE PRESENTATION

**Fig 1:**
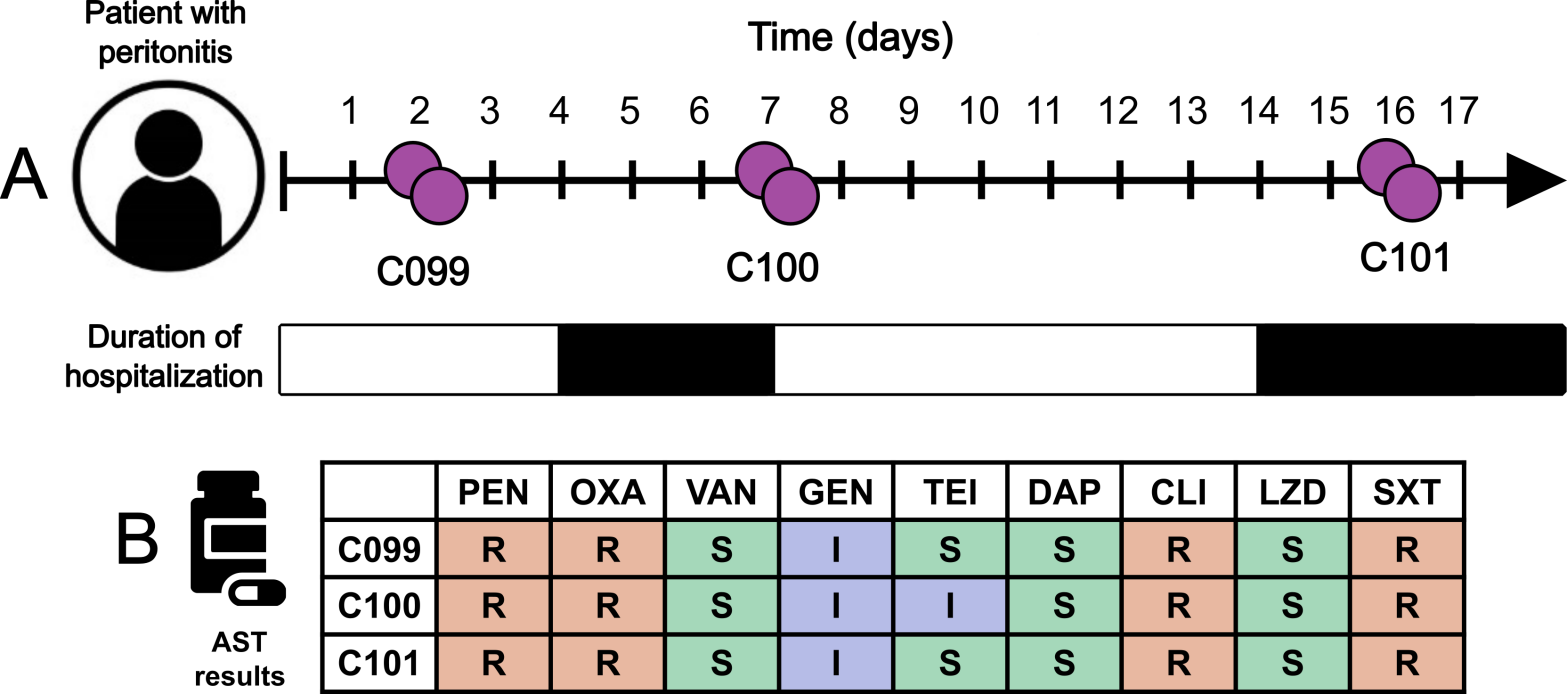
Isolation of MDR *S. epidermidis* isolates from a peritonitis case. (**A**) Timeline of case presentation. Bacterial isolates are shown in purple, and the duration of hospitalization is shaded in black. (**B**) Clinical AST results using the VITEK 2 system. Benzylpenicillin, PEN; oxacillin, OXA; vancomycin, VAN; gentamicin, GEN; ciprofloxacin, CIP; clindamycin, CLI; linezolid, LZD; trimethoprim/sulfamethoxazole, SXT. Results shown as resistant (red), susceptible (green), or intermediate (blue) inferred from breakpoints in CLSI M100 27th ed (17). All three clinical isolates tested were susceptible to vancomycin.

In 2017, a 63-year-old male presented to a community peritoneal dialysis center for review of cloudy dialysis effluent (Fig. 1A). Dialysate samples were sent to the clinical laboratory for testing, and empirical treatment commenced with 200 mg of gentamicin and 2 g of vancomycin administered IP (following the statewide peritonitis treatment protocol with a minimum dwell time of 6 hours). Four days later, he presented to the hospital with persistent cloudy effluent and new abdominal pain. *S. epidermidis* was isolated from the samples (ID: C099), and treatment was changed to vancomycin monotherapy with the isolate recorded as susceptible using the VITEK 2 (Fig. 1B; Table 1). On admission, serum vancomycin levels demonstrated that he was within a therapeutic range at 17 mg/L (target >15 mg/L). On his third day of admission, he was improving clinically with no ongoing abdominal pain. A repeat sample was sent with results demonstrating an improvement in white cell count from 5.4 × 10^10^ /L (100% neutrophils) in the initial sample to 1.8 × 10^10^ /L (74% neutrophils). He was discharged with a plan to continue IP vancomycin for 21 days.

**TABLE 1:** MIC values of clinical isolates tested by VITEK 2^*c*^

Isolate ID	PEN	OXA	GEN	CIP	CLI	LZD	DAP	TEI	VAN	TET	RIF	SXT^*a*^
C099	≥0.5 (*R*)	≥4 (*R*)	8 (*I*)	4 (*R*)	≥8 (*R*)	1 (*S*)	1 (*S*)	8 (*S*)	2 (*S*)	2 (*S*)	≤0.5 (*S*)	160 (*R*) (8/152)
C100	≥0.5 (*R*)	≥4 (*R*)	4 (*S*)	≥8 (*R*)	≥8 (*R*)	1 (*S*)	0.5 (*S*)	16 (*I*)	2 (*S*)	2 (*S*)	≤0.5 (*S*)	160 (*R*) (8/152)
C101	N/A^*b*^	N/A	N/A	N/A	N/A	N/A	N/A	N/A	N/A	N/A	N/A	N/A

^*a*^ VITEK 2 SXT MICs are reported as the sum of trimethoprim and sulfamethoxazole MICs (1:19 ratio).

^*b*^ VITEK 2 MIC results were not available.

^*c*^ Abbreviations and test reference ranges (µg/mL): benzylpenicillin, PEN (0.03–5); oxacillin, OXA (0.25–4); gentamycin, GEN (0.5–16); ciprofloxacin, CIP (0.5–8); clindamycin, CLI (0.25–8); linezolid, LZD (0.5–8); daptomycin, DAP (0.12–8); teicoplanin, TEI (0.5–32); vancomycin, VAN (0.5–32); tetracycline, TET (1–16); rifampicin, RIF (0.5–32); trimethoprim/sulfamethoxazole, SXT (10-320). Result interpretations (in italics): resistant (R), intermediate (I), and susceptible (S). Results were inferred using break-point tables available from CLSI M100 27th Edition (41).

On day 7, *S. epidermidis* was again cultured from the effluent samples (ID: C100). On day 14 (after initial presentation), the patient presented to the hospital again with abdominal pain and fluid overload. Serum vancomycin levels remained within the therapeutic range, and repeat microscopy continued to show an improvement in white cell counts: 2.5 × 10^9^ /L (70% neutrophils). Dialysate samples (day 16), however, remained culture positive for *S. epidermidis* (ID: C101). All isolates (C099, C100, and C101) were susceptible to vancomycin according to the VITEK 2 (Fig. 1B; Table 1). As the patient could no longer adequately dialyze, the Tenckhoff catheter was removed (17 days after his initial presentation), and the patient was transferred to hemodialysis.

To investigate the potential causes for failure to resolve infection, we performed additional AST using the BMD method with Sensititre custom plates (Thermo Fisher, United States), with results shown in Table 2. The clinical isolates were susceptible to vancomycin (MIC = 2 µg/mL) and other antimicrobials, including cefoxitin (MIC = 8 µg/mL), teicoplanin (MIC = 4 µg/mL), daptomycin (MIC = 0.5 µg/mL), and linezolid (MIC ≤0.5 µg/mL). Isolates were resistant to benzylpenicillin (MIC = ≥1 µg/mL), oxacillin (MIC = 2 µg/mL), trimethoprim/sulfamethoxazole (MIC = 8 µg/mL), and clindamycin (MIC ≥2 µg/mL). VITEK 2 MIC results were not recorded for C101; however, our BMD MIC results for vancomycin and other antimicrobials tested were concordant or within essential agreement (EA) with the clinical laboratory results, with the exception of MIC results for teicoplanin for C100 (discrepancy just outside of EA). Isolates were defined as MDR as they were resistant to multiple antimicrobials from more than three antimicrobial classes (18).

**TABLE 2:** MIC values of clinical isolates tested by BMD^*b*^

Isolate ID	P/T	PEN	OXA	FOX	VAN	TEI	GEN	SXT^*a*^	DAP	CLI	AMO	LZD	AXO
C099	2 (*N/A*)	≥1 (*R*)	2 (*R*)	8 (*S*)	2 (*S*)	4 (*S*)	8 (*I*)	8 (*R*)	0.5 (*S*)	≥2 (*R*)	2 (*N/A*)	≤0.5 (*S*)	8 (*N/A*)
C100	1 (*N/A*)	≥1 (*R*)	2 (*R*)	8 (*S*)	2 (*S*)	4 (*S*)	8 (*I*)	8 (*R*)	0.5 (*S*)	≥2 (*R*)	2 (*N/A*)	≤0.5 (*S*)	8 (*N/A*)
C101	2 (*N/A*)	≥1 (*R*)	2 (*R*)	8 (*S*)	2 (*S*)	4 (*S*)	8 (*I*)	8 (*R*)	0.5 (*S*)	≥2 (*R*)	2 (*N/A*)	≤0.5 (*S*)	8 (*N/A*)

^*a*^ BMD MICs for SXT are reported as trimethoprim MIC in microgram per milliliter.

^*b*^ Abbreviations and test reference ranges (µg/mL): piperacillin/tazobactam, P/T (0.25–4); benzylpenicillin, PEN (0.03–1); oxacillin, OXA (0.12–4); cefoxitin, FOX (1–8); vancomycin, VAN (0.12–16); teicoplanin, TEI (0.5–16); gentamycin, GEN (0.5–8); trimethoprim/sulfamethoxazole, SXT (0.5–32); daptomycin, DAP (0.25–4); clindamycin, CLI (0.12–2); amoxicillin, AMO (0.25–4); linezolid, LZD (0.5–8); and ceftriaxone, AXO (0.12–8). Result interpretations (in italics): resistant (R), intermediate (I), susceptible (S), and no break-points available (N/A). Results were inferred using break-point tables available from CLSI M100 27th Edition (41).

**Fig 2:**
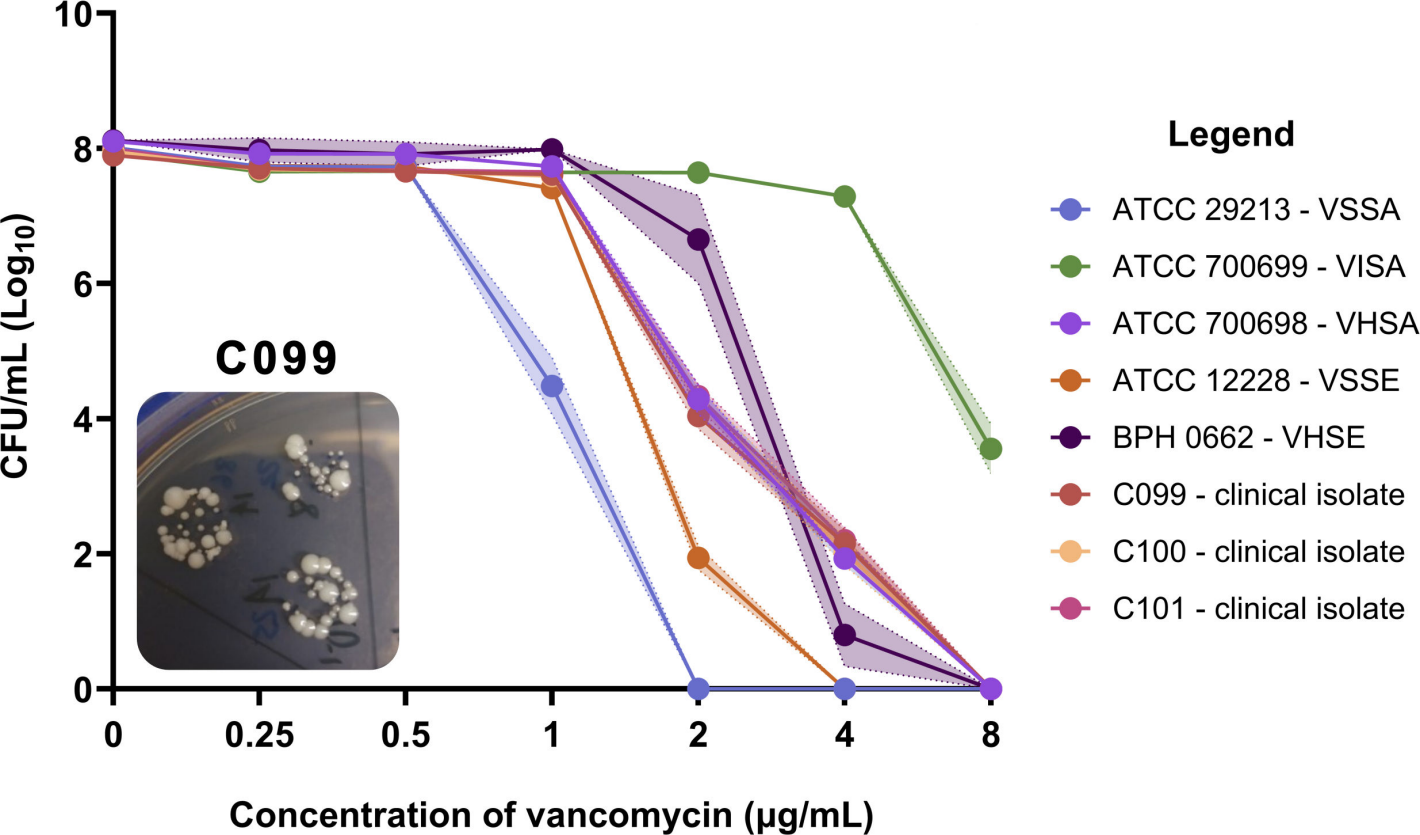
Confirmation of heteroresistance using the PAP-AUC method. Shaded bars indicate SEM. VSSA, vancomycin susceptible *S. aureus* (blue); VISA, vancomycin intermediate *S. aureus* (15); VHSA, vancomycin heteroresistant *S. aureus* (purple); VSSE, vancomycin susceptible *S. epidermidis* (orange); VHSE, vancomycin heteroresistant *S. epidermidis* (dark purple); C099 (red); C100 (cream); and C101 (pink). Image of C099 growing on 2 µg/mL of vancomycin (10^−1^ dilution). Heterogeneous colony phenotypes (small colony variants) were seen in all three clinical isolates growing in 2 and 4 µg/mL of vancomycin.

Heteroresistance is detected using the “gold-standard” population analysis profiling area under the curve (PAP-AUC) method (12) with test isolates compared to ATCC 700698, a reference control *Staphylococcus aureus* with documented heterogeneous resistance to vancomycin (19). Heteroresistance was confirmed if the area under the curve (AUC) of the test isolate divided by ATCC 700698 was ≥0.90 (20). While previous studies have applied this method directly to CoNS isolates (21), we included two *S*. *epidermidis* controls: ATCC 12228 (vancomycin susceptible) and BPH0662 (vancomycin heteroresistant) to further investigate the applicability of the diagnostic criteria to *S. epidermidis*. AUC ratios of 0.60 and 1.02 were obtained, respectively, for the controls, confirming classification validity, and all three clinical isolates were confirmed as heteroresistant to vancomycin (Fig. 2), with AUC ratios of 1.00, 1.00, and 1.03 for C099, C100, and C101 recorded. The sequential isolates C099, C100, and C101 also displayed small colony variant phenotypes growing on brain heart infusion (BHI) agar plates containing vancomycin (Fig. 2) and on blood agar plates.

In addition to phenotypic characterisation, WGS was also performed using both Illumina NextSeq (Illumina, United States) and MinION (Oxford Nanopore Technologies, United Kingdom) to investigate the genetic basis for the heteroresistance. All three isolates were nearly genetically identical (OrthoANIu percentages of 99.99% with over 99% genome coverage) (22). The resistome of each isolate was identified using Resfinder 4.1 (23), ABRicate (Galaxy Version 1.0.1 using the public server at usegalaxy.org.au) (24), and Comprehensive Antibiotic Resistance Database (CARD) (25). Each isolate harbored multiple chromosomal and plasmid-encoded resistance genes (Table 3) but did not harbor *van* genes associated with vancomycin resistance (26). The three isolates were screened for mutations in genes previously associated with vancomycin heteroresistance (Table 4). We were unable to detect any single-nucleotide polymorphisms in candidate genes associated with vancomycin heteroresistance, such as dual D471E/I527M mutations in the *rpoB* gene, the mechanism for heteroresistance in BPH0662 (27).

**TABLE 3:** Genomic characteristics of *S. epidermidis* isolates

Isolate ID	Genome characteristics		Genetic resistance markers^*a*^						
	Size (bp)	CDS^*b*^	*blaZ*	*dfrG*	*fosB*	*mupA*	*mecA*	*vga(A*)	*aac(6')-aph(2''*)
C099	2,490,698	2,257	+	+	+	+	+	+	+
C100	2,489,174	2,267	+	+	+	+	+	+	+
C101	2,489,413	2,260	+	+	+	+	+	+	+

^*a*^ Antibiotic resistance markers encoded on the chromosome are shaded in gray, while markers encoded on plasmids are shaded in white.

^*b*^ CDS, coding sequence.

**TABLE 4:** Mutations associated with vancomycin heteroresistance in *S. aureus* and *S. epidermidis*

Gene	Mutation^*b*^	Present in isolates?
*rpoB*	H481Y (28)	No
	D471E (29)	No
	I527M (29)	No
	A621E (30)	No
	N651I (14)	No
	P940S (14)	No
*walK*	G223D (31)	No—S223^*a*^
	I237T (32)	No—D237^*a*^
	I306F (14)	No
	R286 (14)	No
*graR*	N197 (33)	No
*graS*	T136I (34)	No
*clpP*	144 bp deletion (35)	No

^*a*^ Actual amino acids present at that location in the gene are listed. Although different from *S. aureus*, the amino acids listed in that location are present in ATCC 12228 (vancomycin-susceptible control *S. epidermidis* strain).

^*b*^ Mutations discovered in *S. epidermidis* are highlighted in gray.

## DISCUSSION

We document a case of vancomycin treatment failure in a patient with MDR *S. epidermidis* peritonitis where conventional AST failed to identify isolates as being resistant, and we subsequently confirmed heteroresistance. Heteroresistance was only detected using the “gold-standard” PAP-AUC method (12), and a genetic basis of the mechanism driving this phenotype was not identified. Heteroresistance is an established phenomenon, occurring in different bacterial species (12, 36), and heterogeneity to vancomycin has been observed in two *S*. *epidermidis* peritonitis cases (37, 38), though not confirmed using the PAP-AUC method. The presence of small colony variants, as documented in our study, has also been associated with the development of heteroresistance in *Escherichia coli* (39) and implicated in chronic and recurrent staphylococcal infections (40).

The choice of antimicrobial treatment for peritonitis relies on results from ASTs; however, there are many limitations to existing tests. While the BMD remains the gold-standard method for susceptibility testing (41), there are detection limitations inherent in BMD assays, as heteroresistant bacteria often have a slower growth rate (42). For staphylococci in particular, heteroresistant subpopulations may also only occur at a frequency of 1 in 10^6^ CFU/mL (43). The limitations of ASTs to detect heteroresistance have also been recently highlighted for the novel antibiotic cefiderocol in cases of carbapenem-resistant *Acinetobacter baumannii* in multiple studies (41, 44). While advances have been made in WGS, the genetic mechanisms of heteroresistance are complex and remain largely cryptic, especially for CoNS (14). To further understand these mechanisms, future studies need to expand the number of complete genomic databases for CoNS, as heteroresistance has been characterized in *S. aureus* to a greater extent. Limitations of this study stem from the nature of conventional antimicrobial susceptibility testing. As these tests measure antimicrobial activity *in vitro*, we cannot fully evaluate the effects of the host environment on infection clearance or the pharmacokinetics of the antimicrobial agents (45). In addition, the catheter from the patient was not available to assess for evidence of colonization or biofilm formation. Biofilm formation has been implicated in increased bacterial resistance (21, 46, 47) and has also been associated with treatment failure (48, 49). While biofilm formation and effective source control are important contributing factors to treatment failure, clinicians also need to be aware of heteroresistance and its implications on patient outcomes.

In conclusion, vancomycin heteroresistance was not detected by conventional AST methods, with the patient failing to clear the infection. Our investigation did not find mutations in genes previously associated with heteroresistance in CoNS isolates C099, C100, and C101; however, the phenotypic PAP-AUC results unequivocally indicated heteroresistance. In the treatment of intractable or non-resolving peritonitis, it is important to recognize the limitations of conventional AST methods and the potential role of heteroresistance, so that therapy can be tailored to prevent adverse outcomes. Our results highlight the need for standardized, rapid screening assays that could be utilized at clinical laboratories to detect heteroresistance and help guide appropriate antimicrobial therapy, ultimately improving patient outcomes.

**TABLE 5:** Accession numbers of isolate chromosomes and plasmids

Isolate number	Local ID	Accession
C099	C099 chromosome	CP094859
	pC99MK1	CP094860
	pC99MK2	CP094861
	pC99MK3	CP094862
	pC99MK4	CP094863
	pC99MK5	CP094864
C100	C100 chromosome	CP094865
	pC100MK1	CP094866
	pC100MK2	CP094867
	pC100MK3	CP094868
	pC100MK4	CP094869
	pC100MK5	CP094870
	pC100MK6	CP094871
C101	C101 chromosome	CP094872
	pC101MK1	CP094874
	pC101MK2	CP094873
	pC101MK3	CP094875
	pC101MK4	CP094876
	pC101MK5	CP094877
	pC101MK6	CP094878

## Data Availability

Nucleotide sequences of C099, C100, and C101 (accession numbers shown in Table 5) are available in the GenBank database (Bioproject number: PRJNA821968). Additional data may be provided upon reasonable request.
